# Complete Genome Sequence of Canine Papillomavirus Type 11

**DOI:** 10.1128/genomeA.00529-14

**Published:** 2014-05-29

**Authors:** Dan Zhou, Jennifer Luff, Yukari Usuda, Verena Affolter, Peter Moore, Richard Schlegel, Hang Yuan

**Affiliations:** a Department of Pathology, Georgetown University Medical School, Washington, DC, USA; b Department of Pathology, Microbiology and Immunology, School of Veterinary Medicine, University of California, Davis, Davis, California, USA

## Abstract

Papillomaviruses with the features of epitheliotropic, nonenveloped, circular, and double-stranded DNA belong to the family *Papillomaviridae*, which contributes to benign and malignant tumors in humans and animals. We report the whole-genome sequence of canine papillomavirus type 11 found at a pigmented plaque located on the skin of a mixed-breed bloodhound.

## GENOME ANNOUNCEMENT

Members of the *Papillomaviridae* family have a genome composed of circular double-stranded DNA, with a length of about 8,000 nucleotides (1). Papillomaviruses (PVs) give rise to a wide spectrum of cutaneous, mucocutaneous, and mucosal proliferations. So far, >100 types have been identified in humans (1, 2).

Canine papillomavirus (CPV) infections generally occur on the head, lips, and legs of young dogs (3–5). Different types of CPVs have been found to be associated with distinct pathologies, including exophytic warts (as in canine oral papillomatosis), endophytic warts, pigmented plaques, and, in some cases, squamous cell carcinomas (3, 6). At present, 10 types of CPV have been fully sequenced (5). The CPV infection model is one of the best systems for studying both epidermal and mucosal PV infections, and the analysis of canine oral papillomavirus type 1 (CPV-1) was critical to understanding host immunity against PV infection (7, 8) and to the human papillomavirus vaccine (9, 10).

This report describes the complete viral genome of a novel CPV type, designated CPV type 11 (CPV-11). Rolling-circle ampliﬁcation (RCA) was used to amplify episomal CPV DNA (11). The ampliﬁed viral genome was cloned into the HindIII site of the vector pUC19 and sequenced using primer walking-enabled sequencing of the entire viral genome from both directions. Analysis of the viral sequence was performed using ABI 3730xl DNA-analyzing instruments (Applied Biosystems) for capillary electrophoresis and ﬂuorescent dye terminator detection. The Vector NTI Advance 10 software (Invitrogen, USA) was used to assemble the sequence contigs containing high-quality trace ﬁles.

The complete genome sequence revealed that CPV-11 is 7,828 bp. Similar to other PVs, CPV-11 has all of its open reading frames (ORFs) on the same coding strand of its circular double-stranded DNA genome. CPV-11 has seven ORFs that encode ﬁve early (E) proteins: E1, E2, E4, E6, and E7. There are two late (L) proteins, L1 and L2. The L1 gene is the most conserved gene within the PV genome and has therefore been used for the identiﬁcation of new PV types. A new PV isolate is recognized if the DNA sequence of the L1 ORF differs by >10% from the closest known PV type (1). The L1 DNA of CPV-11 is most closely related (75% homology) to that of the recently discovered CPV-5. These data will facilitate future investigations of the evolutionary characteristics and molecular pathogenesis of CPVs.

### Nucleotide sequence accession number.

The complete genome sequence of CPV-11 is available in GenBank under the accession no. JF800658.1.
